# On the assembly of the mitotic spindle, bistability and hysteresis

**DOI:** 10.1007/s00018-023-04727-6

**Published:** 2023-03-08

**Authors:** Angela Flavia Serpico, Caterina Pisauro, Domenico Grieco

**Affiliations:** 1 CEINGE Biotecnologie Avanzate Franco Salvatore, Naples, Italy; 2 DMMBM, University of Naples “Federico II”, Naples, Italy

**Keywords:** Spindle assembly, i-Cdk1, Checkpoint, Genome stability, Chromosome segregation, Cell cycle progression

## Abstract

During cell division, the transition from interphase to mitosis is dictated by activation of the cyclin B-cdk1 (Cdk1) complex, master mitotic kinase. During interphase, Cdk1 accumulates in an inactive state (pre-Cdk1). When Cdk1 overcomes a certain threshold of activity upon initial activation of pre-Cdk1, then the stockpiled pre-Cdk1 is rapidly converted into overshooting active Cdk1, and mitosis is established irreversibly in a switch-like fashion. This is granted by positive Cdk1 activation loops and the concomitant inactivation of Cdk1 counteracting phosphatases, empowering Cdk1 activity and favoring the Cdk1-dependent phosphorylations that are required to establish mitosis. These circuitries prevent backtracking and ensure unidirectionality so that interphase and mitosis are considered bistable states. Mitosis also shows hysteresis, meaning that the levels of Cdk1 activity needed to establish mitosis are higher than those required to maintain it; therefore, once in mitosis cells can tolerate moderate drops in Cdk1 activity without exiting mitosis. Whether these features have other functional implications in addition to the general action of preventing backtracking is unknown. Here, we contextualize these concepts in the view of recent evidence indicating that loss of activity of small and compartmentalized amounts of Cdk1 within mitosis is necessary to assemble the mitotic spindle, the structure required to segregate replicated chromosomes. We further propose that, in addition to prevent backtracking, the stability and hysteresis properties of mitosis are also essential to move forward in mitosis by allowing cells to bear small, localized, drops in Cdk1 activity that are necessary to build the mitotic spindle.

## Introduction

Cycles of activation and inactivation of cyclin-dependent protein kinases (CDKs) drive the eukaryotic cell division [1, 2]. In particular, the transition from interphase into mitosis is triggered by activation of the cyclin B-cdk1 (Cdk1) complex [2]. During interphase, the Cdk1 complex accumulates in an inactive state as the cdk1 moiety undergoes phosphorylations, carried out by the Wee1 and Myt1 protein kinases, that preclude the kinase action of the complex (pre-Cdk1) [2]. At the interphase-to-mitosis transition, the Cdc25 phosphatases remove these Cdk1 inhibitory phosphorylations, leading to Cdk1 activation, nuclear accumulation of the complex and the onset of mitosis [2–5]. Upon initial Cdk1 activation, in the absence of stressful conditions, biochemical networks create positive Cdk1 activation loops that rapidly convert stockpiled pre-Cdk1 into active Cdk1, so that the system switches from interphase to mitosis with such a strong directionality that prevents backtracking, almost as a ratchet, creating bistability and hysteresis [2–4, 6]. The return to interphase is only licensed upon completion of spindle assembly and granted by the rapid destruction of Cdk1 by proteolysis of cyclin B [3, 7, 8]. Cyclin B degradation, Cdk1 inactivation and anaphase onset are under the negative control of the spindle assembly checkpoint (SAC), a safeguard mechanism that only gets silenced upon correct bipolar attachment of all replicated chromosome to spindle microtubules [7–9]. The SAC is held by Cdk1 activity itself, in particular, by active Cdk1 localized at unattached or mis-attached kinetochores [7, 9–14]. This dependence is also a major determinant that renders irreversible the mitosis exit program shortly after cyclin B degradation is licensed upon correct bipolar attachment of all chromatid pairs. Indeed, when the SAC is satisfied, cyclin B degradation begins prior to loss of sister chromatid cohesion [15]. This ensures that, upon sister chromatid separation, the loss of kinetochore tension, a condition that would have had activated the SAC during spindle assembly, does not reactivate the SAC during anaphase because Cdk1 activity has already dropped below the levels required to sustain the SAC [16, 17].

## Mitosis: bistability and hysteresis

**Fig. 1:**
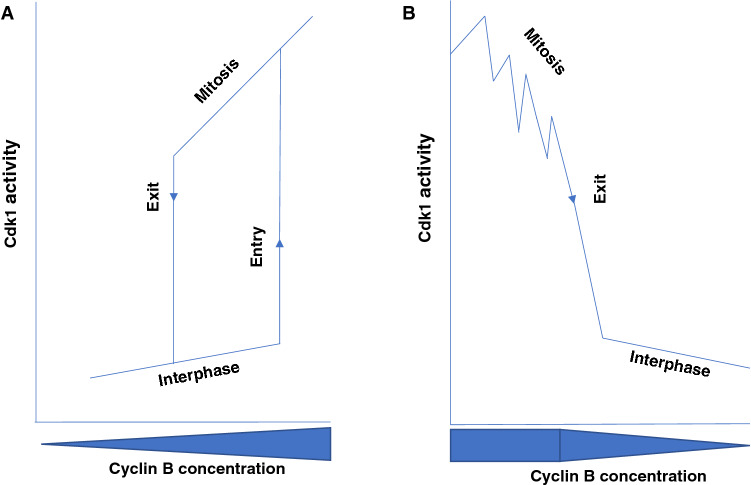
Bistability and hysteresis of interphase and mitosis. **a** The transition from interphase to mitosis is switch-like above an “Entry” cyclin B concentration threshold level. Above the “Entry” cyclin B concentration threshold level, positive feedback loops are ingnited and an overshooting Cdk1 activity establishes mitosis. The transition from mitosis to interphase ensues when cyclin B drops below an “Exit” cyclin B concentration threshold level. Note that the “Entry” threshold of cyclin B concentration is higher than the “Exit” threshold, this helps preventing backtracking. **b** The level of Cdk1 activity reached in mitosis is significantly higher than the “Exit” threshold level of Cdk1 activity, below which the system returns to interphase. Thus, fluctuations in Cdk1 activity above the “Exit” threshold can be absorbed without exiting mitosis. These bistabilty and hysteresis features allow inhibitory control of Cdk1 via i-Cdk1 formation by inhibitory phosphorylation and inhibitory proteins like p21 and Cdc6 that is required for the assembly of the mitotic spindle and mitosis progression

At the onset of mitosis, upon the initial trigger, the rapid Cdk1 activation ensues from positive feedback loops by which Cdk1 directly and indirectly stimulates Cdc25 and inhibits Wee1/Myt1 activities [2–4]. In addition, Cdk1 directly and indirectly inhibits the action of major phosphatases like PP1 and PP2A-B55 that would, otherwise, antagonize the Cdk1-positive loops and other Cdk1-dependent phosphorylations needed to establish mitosis [2, 18, 19]. These circuitries contribute to strengthen the unidirectionality of the interphase-to-mitosis transition and the stability and hysteresis of the mitotic state [3]. By titrating amounts of cyclin B required to activate Cdk1 in interphase Xenopus egg extracts, in pioneering experiments, Solomon et al. showed that entry into mitosis did not progress linearly with increasing amounts of cyclin B but, rather, entry into mitosis was triggered switch-like above a certain cyclin concentration threshold level, demonstrating bistability of the interphase and mitosis states (Fig. 1a) [20]. The stability property led to the prediction that mitosis would also show hysteresis: the cyclin concentration threshold to get into mitosis is higher than the threshold to exit from mitosis and that less Cdk1 activity is required to maintain the mitotic state relatively to the amount of enzyme activity needed to establish it (Fig. 1a, b) [21]. This prediction was experimentally confirmed again using the Xenopus egg extract system [22, 23]. Hysteresis was later shown to be reinforced also by the direct and indirect inhibition by Cdk1 of Cdk1-antagonizing phosphatases [18, 19]. These features are certainly important for the correct execution of mitosis since they create a ratchet type of mechanism that resists backtracking (Fig. 1a, b). In addition, the stability and hysteresis features also clearly imply that, in mitosis, cells may absorb partial drops in Cdk1 activity while remaining fully in mitosis, thus, maintaining a round up morphology, condensed chromosomes, no nuclear membrane, a cytoplasm essentially free of microtubules, etc. (Fig. 1a, b). The hysteresis feature may allow, for instance, the fact that the cyclin A–cdk1 complex, that also contributes to the overall cdk1 activity at the onset of mitosis, is substantially inactivated by cyclin A degradation before chromosome alignment to the spindle equator, thus generating a partial drop in cdk1 activity while cells are fully in mitosis assembling the mitotic spindle [24].

Could the bistability and hysteresis features also serve other goals by permitting modulation of Cdk1 activity in mitosis?

## Cytoskeletal changes in mitosis and the control of Cdk1 activity

When cells enter mitosis, they must dismantle the intricate interphase microtubular network to prevent chromosome damage during spindle assembly, when chromosomes move around to be finally aligned at the spindle equator. Cdk1 activity is required for these cytoskeletal changes by, for instance, phosphorylating and inhibiting activity of microtubule-associate proteins (MAPs) that stabilize interphase microtubules (MTs) [25–27]. Paradoxically, activity of some these same MAPs is required for spindle assembly and spindle MT stability, despite the high Cdk1 activity in mitosis [27, 28]. Approaching this conundrum, we have recently uncovered that a small amount of Cdk1 remains inhibited by phosphorylation in mitosis (i-Cdk1 for inhibited/inactive Cdk1) [29]. In addition, we found that i-Cdk1 does not localize in the cytoplasm of mitotic cells, but it is selectively bound to spindle structures where it increases during spindle assembly [29]. Antagonizing i-Cdk1 formation in cells, by downregulating Wee1 expression or overexpressing an inhibitory phosphorylation-resistant Cdk1 mutant version, substantially impairs spindle assembly [29]. We found that i-Cdk1 binds active PP1 and, mechanistically, provided evidence that this complex serves for localized reversal of inhibitory phosphorylations of MT-stabilizing MAPs to promote spindle assembly. Moreover, in mitotic cells that were unable to build spindle because of Wee1 downregulation by small interfering RNAs (siRNAs), spindle assembly was restored by mild Cdk1 activity inhibition, by adding low doses of the selective Cdk1 chemical inhibitor RO3306 (500 nM). Upon RO3306 addition, the i-Cdk1 content was restored by recruitment of residual Wee1, that escaped siRNA-mediated downregulation, at the reassembled spindles, meanwhile bulk Cdk1 remained fully dephosphorylated at inhibitory sites and active in the cytoplasm. Thus, our data indicate that in subcellular compartments, like spindle structures, the Cdk1 activity control may evade positive feedback loops, permitting localized loss of Cdk1 activity that is required for spindle assembly. This is possible thanks to the bistability and hysteresis properties of mitosis that allow to bear localized drops in Cdk1 activity, while the rest of Cdk1 remains largely active in the cytoplasm and in other locations, underpinned by positive feedback loops, holding mitosis (Fig. 1b).

## Other evidence for negative control of Cdk1 activity in mitosis

In addition to inhibitory phosphorylation of Cdk1, evidence has been provided that other means of inhibitory control of Cdk1 activity, within mitosis, contribute to the correct execution of mitosis itself. The Cdc6 protein has a crucial role in S phase by licensing origins for DNA replication, but it is also an inhibitor of Cdk1 and has been shown to bind and inhibit Cdk1 in mitosis in a Plk1-dependent manner [30]. Moreover, Cdc6 localized to spindle structures and interfering with the Plk1-Cdc6-Cdk1 axis perturbed chromosome segregation and other aspects of mitosis [30]. More recently, by studying potential causes of chromosomal instability often found in cancer cells and associated with the loss of function of crucial tumor suppressor genes like p53 and p73, Schmidt et al. demonstrated that defects in spindle assembly and in chromosome segregation were caused by loss of p53/p73-dependent induction of the Cdk1 inhibitory protein p21 [31]. The protein p21 is also considered a tumor suppressor gene and its expression if often downregulated in cancer [32]. The authors also showed that loss of p21 per se induced spindle assembly alterations and chromosome segregation errors because of reduced negative control of Cdk1 activity in mitosis [31]. Moreover, spindle abnormalities and segregation error in p21-deficient cells were corrected by treatment with low doses of the Cdk1 inhibitor RO3306 [31]. These data represent other important evidence that hysteresis is crucial to tolerate reductions of Cdk1 activity in mitosis that are needed for the correct execution of mitosis itself.

## Discussion

Mathematical modeling and experimental data have helped to establish that interphase and mitosis are two stable states [3, 4, 19, 21–23]. Indeed, interphase is stable below a certain threshold level of cyclin B concentration [21–23]. Above the threshold, an overshooting Cdk1 activity rapidly ensues, and a stable mitotic state is switched on [21–23]. This bistable condition also implies hysteresis: the amount of Cdk1 activity needed to establish mitosis is higher than that required to maintain it (Fig. 1) [21–23]. These features, based on feedback loops that promote Cdk1 activity and antagonize Cdk1-counteracting phosphatases, are certainly crucial to prevent backtracking of the system and ensure unidirectionality of the cell division cycle, so that chromosome segregation necessarily follows DNA replication [3, 4, 18, 19]. However, recent evidence suggests that stability and hysteresis are also essential features to move forward in mitosis [29–31]. Indeed, cells in mitosis must bear limited and compartmentalized drops in Cdk1 activity to correctly build the mitotic spindle to ensure chromosome segregation and completion of mitosis (Fig. 1b) [29–31]. This fundamental inhibitory control of Cdk1 activity takes place while cells are in mitosis; thus, while the positive feedback loops maintain the rest of Cdk1 active in the cytoplasm and at specific locations, like kinetochores, to hold the SAC and prevent mitosis exit [10–14]. How i-Cdk1 escapes the Cdk1-positive feedback loops and possibly, locally, coexist with active Cdk1 is unknown at present. Cdk1 activation begins at centrosomes and it is known that there the DNA damage/replication checkpoint kinase Chk1 coordinates S-phase completion with the initiation of mitosis by inhibiting the Cdc25 phosphatases from dephosphorylating and activating Cdk1 at centrosomes until S-phase completion [33, 34]. However, it has also been reported that, even after Cdk1 activation is licensed, active forms of Chk1 persist in mitosis and are localized at spindle microtubules during spindle assembly [35, 36]. Moreover, downregulating Chk1 in mitosis causes profound defects in spindle assembly, chromosome segregation and cytokinesis [35]. Thus, an experimentally testable hypothesis is that spindle-localized Chk1 may contribute to locally inhibit Cdc25, antagonizing the Cdk1-positive loops and favoring localized maintenance and formation of i-Cdk1 for spindle assembly.

Based on these considerations, we would like to propose that the stability and hysteresis properties of mitosis, in addition to prevent backtracking to interphase, are essential to move forward in mitosis by allowing cells to bear localized losses of Cdk1 activity that are necessary to build the mitotic spindle, the structure needed to accomplish the goal of mitosis, chromosome segregation.

## Data Availability

Enquiries about data availability should be directed to the authors.
